# Persistent transmission of *Plasmodium malariae* and *Plasmodium ovale* species in an area of declining *Plasmodium falciparum* transmission in eastern Tanzania

**DOI:** 10.1371/journal.pntd.0007414

**Published:** 2019-05-28

**Authors:** Victor Yman, Grace Wandell, Doreen D. Mutemi, Aurelie Miglar, Muhammad Asghar, Ulf Hammar, Mattias Karlsson, Ingrid Lind, Cleis Nordfjell, Ingegerd Rooth, Billy Ngasala, Manijeh Vafa Homann, Anna Färnert

**Affiliations:** 1 Division of Infectious Diseases, Department of Medicine Solna, Karolinska Institutet, Stockholm, Sweden; 2 Department of Otolaryngology, University of Washington Medical Center, Seattle, Washington, United States of America; 3 Department of Parasitology and Medical Entomology, Muhimbili University of Health and Allied Sciences, Dar es Salaam, Tanzania; 4 Unit of Biostatistics, Department of Environmental Medicine, Karolinska Institutet, Stockholm, Sweden; 5 Department of Women’s and Children’s Health, International Maternal and Child Health (IMCH), Uppsala University, Uppsala, Sweden; 6 Department of Infectious Diseases, Karolinska University Hospital, Stockholm, Sweden; International Vaccine Institute, REPUBLIC OF KOREA

## Abstract

A reduction in the global burden of malaria over the past two decades has encouraged efforts for regional malaria elimination. Despite the need to target all *Plasmodium* species, current focus is mainly directed towards *Plasmodium falciparum*, and to a lesser extent *P*. *vivax*. There is a substantial lack of data on both global and local transmission patterns of the neglected malaria parasites *P*. *malariae* and *P*. *ovale* spp. We used a species-specific real-time PCR assay targeting the *Plasmodium* 18s rRNA gene to evaluate temporal trends in the prevalence of all human malaria parasites over a 22-year period in a rural village in Tanzania.We tested 2897 blood samples collected in five cross-sectional surveys conducted between 1994 and 2016. Infections with *P*. *falciparum*, *P*. *malariae*, and *P*. *ovale* spp. were detected throughout the study period, while *P*. *vivax* was not detected. Between 1994 and 2010, we found a more than 90% reduction in the odds of infection with all detected species. The odds of *P*. *falciparum* infection was further reduced in 2016, while the odds of *P*. *malariae* and *P*. *ovale* spp. infection increased 2- and 6-fold, respectively, compared to 2010. In 2016, non-falciparum species occurred more often as mono-infections. The results demonstrate the persistent transmission of *P*. *ovale* spp., and to a lesser extent *P*. *malariae* despite a continued decline in *P*. *falciparum* transmission. This illustrates that the transmission patterns of the non-falciparum species do not necessarily follow those of *P*. *falciparum*, stressing the need for attention towards non-falciparum malaria in Africa. Malaria elimination will require a better understanding of the epidemiology of *P*. *malariae* and *P*. *ovale* spp. and improved tools for monitoring the transmission of all *Plasmodium* species, with a particular focus towards identifying asymptomatic carriers of infection and designing appropriate interventions to enhance malaria control.

## Author summary

The reduction in the global burden of malaria has encouraged efforts for elimination. Attempts to control and monitor transmission have mainly focused on the predominant malaria parasites *Plasmodium falciparum* and *P*. *vivax*. However, eliminating malaria requires the elimination of all human malaria parasites and limited interest has been directed towards estimating the disease burden attributable to the neglected malaria parasites *P*. *ovale* spp. and *P*. *malariae*. The authors used molecular methods to analyse 2897 blood samples collected in five cross-sectional surveys over a period of 22 years, and described the transmission patterns of all human malaria parasites in a Tanzanian village. They demonstrate a persistent transmission of *P*. *malariae* and *P*. *ovale* spp. despite a substantial reduction in transmission of *P*. *falciparum*, highlighting the need for more attention towards non-falciparum malaria. The authors discuss the implications of these findings in the context of current efforts for regional malaria elimination.

## Introduction

Since the turn of the millennium, there has been a substantial reduction in the global burden of malaria including a reduction in the clinical incidence of both *Plasmodium falciparum* and *P*. *vivax* malaria [1–3]. This reduction has largely been attributed to an increase in malaria control efforts using insecticide treated nets (ITNs), indoor residual spraying (IRS), improved diagnostics through the use of rapid diagnostic tests (RDTs), and better access to highly efficacious artemisinin based combination therapy (ACT) [2,4]. Several countries are approaching a hypoendemic or unstable transmission setting and in 2016 the World Health Organization (WHO) identified 21 countries in which malaria elimination was deemed feasible by the year 2020 [3].

The focus of malaria control programmes has historically mainly been directed towards limiting transmission of *P*. *falciparum* and to a lesser extent also of *P*. *vivax*. However, achieving malaria elimination requires the elimination of all malaria parasites infecting humans (i.e. *P*. *falciparum*, *P*. *vivax*, *P*. *malariae*, and *P*. *ovale curtisi* and *wallikeri* as well as the simian species, e.g. *P*. *knowlesi* in South East Asia) [3,5,6]. While *P*. *malariae* and *P*. *ovale* spp. are reported to be widely distributed throughout tropical Africa and other malaria endemic regions of the world, their epidemiology remains far less studied than that of *P*. *falciparum* and *P*. *vivax* and both global and local temporal trends in transmission intensity are largely unknown [7–10].

Although generally considered benign, *P*. *malariae* and *P*. *ovale* spp. have the potential to cause significant morbidity. Infection with *P*. *malariae* is an established cause of nephrotic syndrome, which can lead to progressive renal failure, particularly in adolescents or young adults [11,12] and has been associated with a high burden of anaemia [13]. Furthermore, *P*. *ovale* spp. has in recent years been recognised as a potential cause of severe malaria [14–16].

In malaria endemic areas of tropical Africa, the majority of clinical malaria attacks are attributed to *P*. *falciparum* [17]. This is partly due to an under-diagnosis of non-falciparum infections. Detection of infection and accurate discrimination of *Plasmodium* species using microscopy requires both highly skilled microscopists and good quality microscopes. It is particularly difficult in asymptomatic low density infections, or mixed species infections with *P*. *falciparum*, in which both *P*. *malariae* and *P*. *ovale* spp. frequently occur [7,18,19]. In addition, RDTs which are currently used as an important diagnostic tool in many settings, have shown poor performance for the detection of *P*. *malariae* and *P*. *ovale* spp. [20–23]. Given the potential to cause morbidity in combination with the under-diagnosis of non-falciparum malaria in many settings, it is likely that the global disease burden attributable to *P*. *malariae* and *P*. *ovale* spp. is largely underestimated.

Over the past decade, cross-sectional studies using PCR for parasite detection have generated evidence that the prevalence of both *P*. *malariae* and *P*. *ovale* spp. is greater than was previously reported [5,7]. These surveys usually find *P*. *malariae* to be more common than *P*. *ovale* spp. and have estimated the prevalence to range from 1 to 35% and 1 to 25%, respectively, depending on transmission setting [24–29]. Although a large number of longitudinal studies from sub-Saharan Africa have reported gradual reductions in the prevalence of *P*. *falciparum*, there have been few studies, and none using PCR, that investigate how the prevalence of *P*. *malariae* and *P*. *ovale* spp. change over time as the prevalence of *P*. *falciparum* decreases [2,30].

In this study, we used PCR to evaluate changes in the prevalence of *P*. *falciparum* and non-falciparum infection by analysis of samples collected in five cross-sectional surveys in a Tanzanian village over a period of 22 years. We assessed the temporal trends in prevalence of all human *Plasmodium* spp. in an area experiencing a substantial reduction in the prevalence and transmission of *P*. *falciparum*.

## Material and methods

### Study site and population

The Nyamisati Malaria Research Project was established in 1985 in Nyamisati, a rural fishing village located 150 km south of Dar es Salaam in the Rufiji river delta area in Kibiti District, Tanzania. Malaria transmission in the area is perennial with seasonal fluctuations. Within the project, the same research team conducted repeated cross-sectional surveys between 1986 and 2016 [31]. The surveys consisted of a physical examination including measurement of body temperature, as well as the collection of a venous blood sample and a blood smear. Each participating individual was assigned a unique individual identifier and demographic information (i.e. age, gender and household membership) was collected. The main intervention to reduce malaria transmission in the village was to provide rapid access to diagnosis and antimalarial treatment free of charge. Sulfadoxine-pyrimethamine (SP), alone or in combination with oral quinine, was the first-line treatment from the early 1990’s until ACTs became readily available in the village in 2009. In addition, ITNs were distributed after the surveys in 1993 (300 ITNs to pregnant women and young children) and in 1999 (900 ITNs). Additionally, long-lasting insecticidal nets (LLINs) were distributed after the survey in 2010. The estimated access to bed nets after the surveys was 45% in 1993–1994, 100% in 1999, and approximately 70% in 2010, assuming an average protection of 1.8 individuals per net [32]. Other vector control measures, e.g. indoor residual spraying, have not been used in the village. The study site, the research project, and temporal trends in the transmission of *P*. *falciparum* have been described in previous publications [31,33]. The present study is based on five cross-sectional surveys conducted at the start of the long rainy season (March-May) in 1994, 1995, 1999, 2010, and in 2016. All villagers were invited to participate in these surveys of the Nyamisati population. The final number of sampled individuals varied by survey year but are considered representative of the Nyamisati population, thus at each cross-section including a random selection of individuals with different levels of exposure. In the years when the cross-sectional survey sampling did not equally cover the entire age-range of the population, this was adjusted for in the statistical analyses. The present study included 2897 samples collected from 2005 unique individuals participating in the five cross-sectional surveys. A number of individuals (n = 544) participated in multiple surveys (range: 2–5) over the years, thus contributing 1435 of the total 2897 samples.

### Ethical considerations

The project was approved by the Nyamisati village board and ethical approval was granted by the Ethical Review board of the National Institute for Medical Research in Tanzania, the Regional Ethical Committee at Karolinska Institutet (Dnr. 00–084), and the Regional Ethical Review Board in Stockholm, Sweden (Dnr. 2012/1151–32). In addition, ethical approval for the 2016 survey was granted by the Institutional Review Board at Muhimbili University of Health and Allied Sciences, a delegated activity of the Medical Research Coordinating Committee (MRCC), Tanzania. Oral informed consent was obtained from all study participants and/or their guardians and was documented in a research database. The use of oral consent was approved by the respective ethical review boards and was selected due to a low degree of literacy in the village.

### Real-time PCR for Plasmodium species identification

Venous blood was collected in EDTA, separated, and stored frozen as plasma and packed cells. DNA was extracted from packed cells using Qiagen blood mini kit (Qiagen, Germantown, MD, USA) (1994–1999), a BioRobot M48 Robotic Workstation (Qiagen) (2010), or a magnetic bead separation method using a Hamilton Chemagic Star Robot (Hamilton, Bonadouz, Switzerland) (2016). Real-time PCR was used to qualitatively detect *Plasmodium* infection (*P*. *falciparum*, *P*. *vivax*, *P*. *ovale* spp., and *P*. *malariae*) in the ABI Taqman 7500 or QuantStudio™ 5 Real-Time PCR system (Applied Biosystems, Foster City, CA, USA), following a previously described protocol [34]. The master mix for a single reaction included species-specific probes and forward primers for all four *Plasmodium* species used in combination with a conserved reverse primer. The *P*. *ovale*- and *P*. *malariae*-probes (synthesized by BioSearch Technologies, Novato, CA, USA), and the *P*. *vivax-* and *P*. *falciparum*-probes (synthetized by Applied Biosystems) were each labelled with a distinct fluorophore, and, depending on the master mix, either ROX or Mustang Purple was used as the reference dye [35]. The reaction was performed in a final volume of 25 μl per well containing 5 μl DNA (corresponding to 5 μl of whole blood), 12.5 μl of either TaqMan universal master mix or TaqMan multiplex master mix (Applied Biosystems), 0.5 μl (10 μmol/L) of the *P*. *falciparum*-specific forward primer, 0.125 μl (10 μmol/L) of each of the other species-specific forward primers and 0.5 μl (10 μmol/L) of the reverse primer, 0.2 μl (10 μmol/L) of each species-specific probe, passive reference dye ROX or Mustang Purple and DNA/RNA-free water. The samples were run using a cut-off of 45 cycles to define positive samples, starting with 95 °C for 20 s, followed by the thermal cycles of 95 °C for 1 s and of 60 °C for 20 s. Standards, negative and species-specific positive controls were included on each plate. The assay was optimised to detect all species simultaneously, with a limit of detection of approximately 0.5 parasites per μl blood. The PCR method does not distinguish between the two sympatric species of *P*. *ovale*, i.e. *P*. *ovale curtisi* and *P*. *ovale wallikeri*, but we established that it could detect both of them using serial dilutions of positive controls (kindly provided by Colin Sutherland, LSHTM).

### Data analysis

Data were analysed using R version 3.4.3 (Vienna, Austria. URL https://www.R-project.org) and Stata 14 (StataCorp., College Station, TX, USA). For the purpose of the analyses, a mixed species infection was defined as an infection with *P*. *falciparum* and *P*. *malariae* and/or *P*. *ovale* spp. A non-falciparum infection was defined as an infection with either *P*. *malariae* or *P*. *ovale* spp. or both. Fever at the time of survey was defined as an axillary body temperature of above 37.5 °C and/or a history of fever or “hot body” within 24 hours. Generalized estimating equation (GEE) regression models were used to estimate population-averaged effects while accounting for the statistical dependency of repeated observations from individuals participating in multiple surveys [36]. Multivariable logistic regression models were used to evaluate the prevalence of each of the *Plasmodium* spp. independently over time while adjusting for covariates, i.e. age, sex, and fever at the time of survey. A multinomial logistic regression model was used to jointly evaluate the relative risk ratio of *P*. *falciparum* mono-infections, mixed-species infections, and non-falciparum infections over time while adjusting for the above specified covariates. In all analyses, age was treated as a categorical variable with five categories (<5, 5–8, 9–12, 13–16 and >16 years). *P*-values <0.05 were considered significant.

## Results

### Species-specific infection prevalence

**Fig 1:**
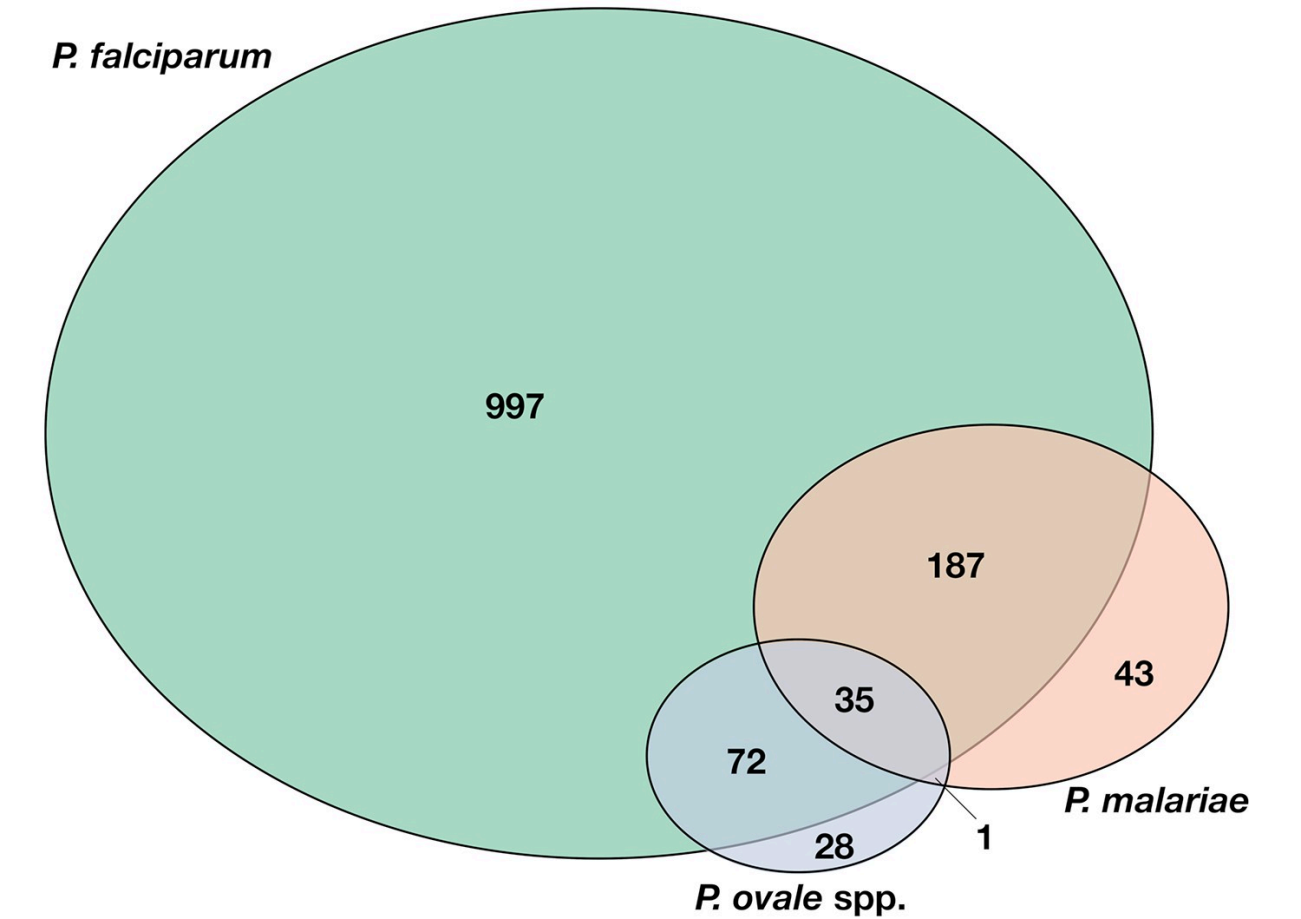
Schematic representation of the number of *Plasmodium* infections detected throughout the five cross-sectional surveys (total number of tested samples n = 2897). The circles indicate number of samples positive for each *Plasmodium* species (Green: *P*. *falciparum*, Red: *P*. malariae, Blue: *P*. *ovale* spp.). The sections where circles overlap represent the number of co-infection of each combination of more than one *Plasmodium* species.

The population characteristics at each of the five cross-sectional surveys are presented in Table 1. Among the total 2897 samples analysed, 1291 (44.5%) were positive for *P*. *falciparum*, 266 (9.2%) for *P*. *malariae*, and 136 (4.7%) for *P*. *ovale* spp. (Fig 1). No samples were positive for *P*. *vivax*. The observed overall parasite prevalence by PCR, including all species, was high during the 1990’s, ranging from 66.1 to 71.6%, but dropped to 19.1% in 2010 and to 17.9% in 2016. *Plasmodium falciparum* was most commonly detected, accounting for 76.3% of positive tests. *Plasmodium malariae* was the second most commonly detected species, found in 15.7% of positive tests, while *P*. *ovale* spp. were detected in 8.0% of positive tests (Fig 1).

**Table 1:** Characteristics of the study site and survey participants.

	1994	1995	1999	2010	2016
**Village population size, n**	1295	1396	1553	n/a	2336
**Cross-sectional survey, n**	792	712	889	808	511
**Subjects in survey with available *real-time* PCR data, n**	596	357	681	752	511
**Female, n (%)**	355 (59.6)	201 (56.3)	365 (53.5)	378 (50.2)	298 (58.1)
**Age, years, median (range)**	18 (0–79)	12 (1–80)	18 (1–84)	15 (1–82)	12 (0–96)
**Children ≤16y, n (%)**	285 (48.2)	235 (65.1)	319 (46.8)	406 (53.9)	396 (77.2)
**Children <5y, n (%)**	74 (12.4)	54 (15.1)	36 (5.3)	47 (6.2)	53 (10.3)
**Fever at time of sampling**^1^	34 (5.9)	42 (11.8)	103 (15.1)	22 (2.9)	96 (18.8)
**Haemoglobin (g/l), mean (range)**	107 (51–163)	109 (50–171)	113 (49–199)	121 (68–188)	120 (66–167)

^1^ Fever at survey defined as body temperature above 37.5°C and/or history of fever or “hot body” within 24 hours.

**Fig 2:**
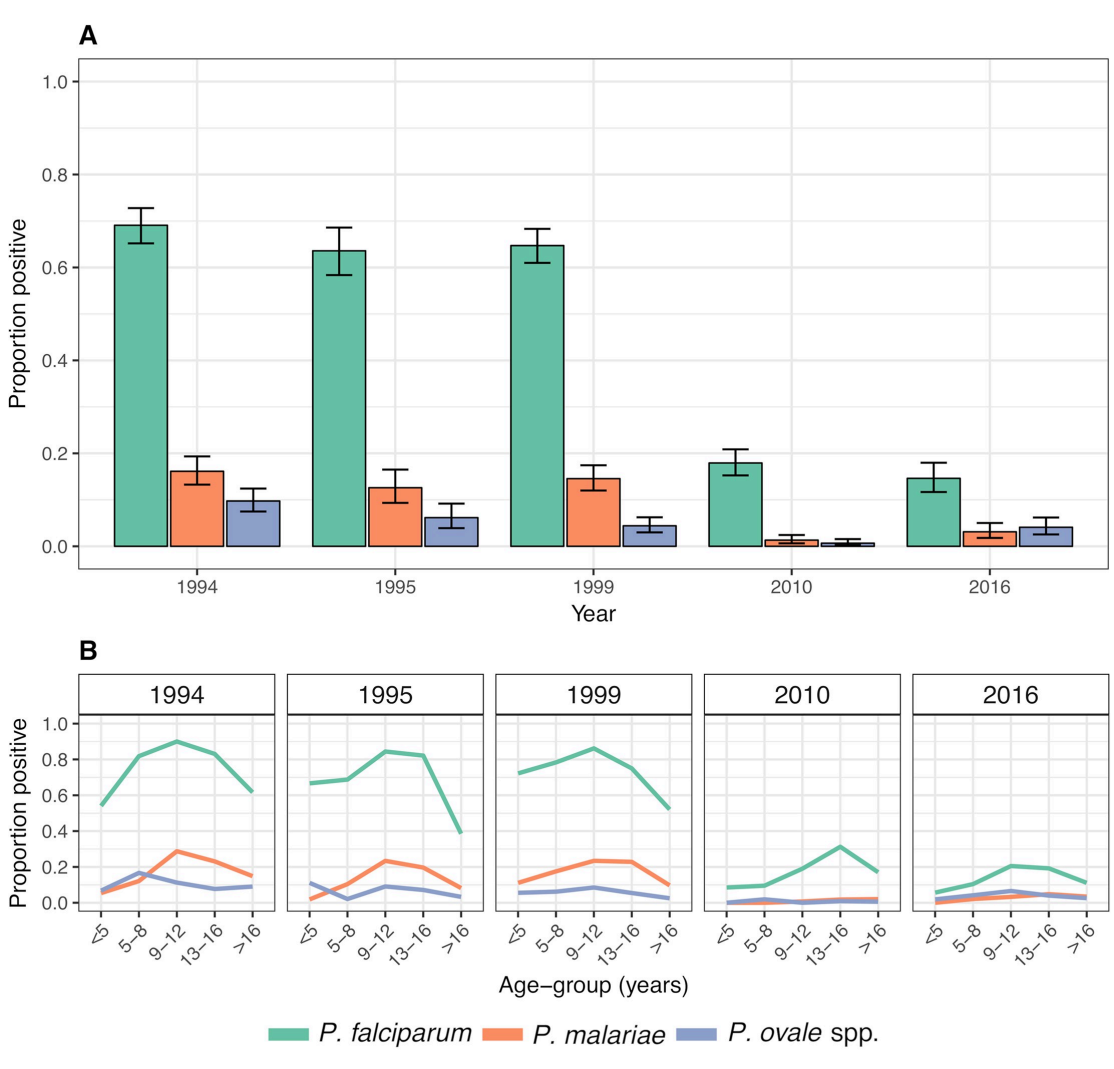
Observed infection prevalence of each *Plasmodium* species. **A)** The observed all-age prevalence of infection with each species of *Plasmodium* in each of the five cross-sectional surveys. The black error bars denote the 95% confidence interval. **B)** The observed age-stratified species-specific infection prevalence in each of the five cross-sectional surveys.

The observed year-wise species-specific prevalence is presented in Fig 2A and stratified by age in Fig 2B. Logistic regression models were used to evaluate the temporal trends in the prevalence of each parasite species independently while adjusting for covariates (i.e. age, sex and fever at the time of survey). The temporal trends are presented as the model-estimated prevalence of infection (with all covariates at their mean values) as well as the corresponding adjusted odds ratios (OR). The logistic regression model estimated a slight decrease in the prevalence of *P*. *falciparum* during the 1990’s, from 73.9% in 1994 to 66.3% in 1999, but the prevalence was markedly reduced thereafter, reaching 17.4% in 2010. The adjusted OR for *P*. *falciparum* infection, comparing 1999 and 2010 to 1994 was 0.70 (95% CI 0.56–0.88; *p =* 0.003) and 0.07 (95% CI 0.06–0.10; *p*<0.001), respectively, i.e. corresponding to a 93% reduction in the odds of infection from 1994 to 2010 (Table 2). Compared to 2010, the prevalence of *P*. *falciparum* infection was further significantly reduced to 10.2% in 2016 (adjusted OR: 0.54; 95% CI 0.39–0.75; *p*<0.001).

**Table 2:** Changes in prevalence of each of the different species over time evaluated using GEE logistic regression models. Adjusted odds ratios of infection with each *Plasmodium* species each survey year compared to base-line 1994.

	*P*. *falciparum*			*P*. *malariae*			*P*. *ovale*		
Year	OR^1^	95% CI	*p*	OR^1^	95% CI	*p*	OR^1^	95% CI	*p*
**1994**	Ref.	-	-	Ref.	-	-	Ref.	-	-
**1995**	0.62	0.47–0.82	0.001	0.68	0.45–0.96	0.031	0.55	0.33–0.93	0.024
**1999**	0.70	0.55–0.88	0.003	0.77	0.57–1.05	0.101	0.42	0.26–0.67	<0.001
**2010**	0.07	0.06–0.10	<0.001	0.06	0.03–0.11	<0.001	0.06	0.02–0.15	<0.001
**2016**	0.04	0.03–0.06	<0.001	0.13	0.07–0.23	<0.001	0.34	0.20–0.58	<0.001

^1^ All odds ratios are adjusted for age (as a categorical variable in five categories: <5, 5–8, 9–12, 13–16, >16), sex and fever at the time of sampling.

The prevalence of *P*. *malariae* remained relatively stable during the 1990’s, with the model-estimated prevalence varying from 11.3% to 16.2%. This was followed by a reduction in the prevalence to 1.1% in 2010 corresponding to a significant 92% reduction in the odds of *P*. *malariae* infection from 1999 to 2010 (adjusted OR: 0.08; 95% CI 0.04–0.15; *p*<0.001) (Table 2). However, in contrast to the further reduction detected for *P*. *falciparum*, there was a significant increase in the prevalence of *P*. *malariae* infection to 2.4% in 2016 (adjusted OR: 2.24; 95% CI 1.01–4.97; *p* = 0.047) (Table 2).

*Plasmodium ovale* spp. were overall least frequently detected with the prevalence of infection declining gradually during the 1990’s, from 10.0% in 1994 to 4.4% in 1999 (adjusted OR: 0.42; 95% CI 0.26–0.67; *p*<0.001). Similar to *P*. *falciparum* and *P*. *malariae*, the prevalence of *P*. *ovale* spp. was further reduced to 0.6% in 2010 (adjusted OR: 0.13, 95% CI 0.05–0.36, *p*<0.001) corresponding to an estimated overall 94% reduction in the odds of infection between 1994 and 2010 (adjusted OR: 0.06, 95% CI: 0.02–0.15, *p*<0.001) (Table 2). However, similarly to *P*. *malariae*, and in contrast to *P*. *falciparum*, there was a subsequent significant increase in the infection prevalence of *P*. *ovale* spp. to 3.6% in 2016 (adjusted OR of 5.9; 95% CI 2.2–15.8, *p*<0.001) compared to 2010 (Table 2), making *P*. *ovale* spp. the second most common infection after *P*. *falciparum* in 2016.

For all species, the observed prevalence was overall highest among 5 to 16 year old children (Fig 2B). A shift of the peak prevalence towards older children was observed for *P*. *falciparum* infection in 2010, but was not as apparent for the other species.

### Mixed species infections

*Plasmodium falciparum* mono-infections represented the majority of infections and accounted for overall 73.1% (95% CI 70.7–75.4%) of infections during the study period (Fig 1). Mixed species infections with *P*. *falciparum* and non-falciparum infections accounted for overall 21.6% (95% CI 19.4–23.9%) and 5.3% (95% CI 4.2–6.6%) of infections, respectively. In *P*. *falciparum* mixed species infections, the combination with *P*. *malariae* was most common, followed by *P*. *ovale* spp., and lastly by infections with all species (Table 3). A non-falciparum infection with both *P*. *malariae* and *P*. *ovale* spp. was detected only once throughout the study period (Table 3).

**Table 3:** Crude relative frequencies of *Plasmodium falciparum* mono- and mixed infections, and non-falciparum infections among positive samples each year of survey.

	1994			1995			1999			2010			2016		
	n	%	95% CI	n	%	95% CI	n	%	95% CI	n	%	95% CI	n	%	95% CI
***Pf* mono**	**289**	**67.7**	**63.0–72.1**	**177**	**75.0**	**70.0–80.4**	**345**	**74.4**	**70.1–78.3**	**128**	**88.8**	**83.4–94.1**	**57**	**62.0**	**51.2–71.9**
***Pf* mixed**	**123**	**28.8**	**24.6–33.4**	**50**	**21.2**	**16.1–27.0**	**97**	**20.9**	**17.3–24.9**	**6**	**4.8**	**1.5–8.8**	**18**	**19.6**	**12.0–29.1**
*Pf*, *Pm*	71	16.6	13.2–20.5	30	12.7	8.7–17.6	73	15.7	12.5–19.4	4	2.8	0.8–7.0	9	9.8	4.6–17.8
*Pf*, *Po*	36	8.4	6.0–11.5	12	5.1	2.7–8.7	15	3.2	1.8–5.3	2	1.4	0.2–4.9	7	7.6	3.1–15.1
*Pf*, *Pm*, *Po*	16	3.7	2.1–6.0	8	3.4	1.5–6.6	9	1.9	0.9–3.7	0	0	0.0–2.5*	2	2.2	0.3–7.6
**Non-Pf**	**15**	**3.5**	**2.0–5.7**	**9**	**3.8**	**1.8–7.1**	**22**	**4.7**	**3.0–7.1**	**9**	**6.3**	**2.9–11.6**	**17**	**18.4**	**11.1–28.0**
*Pm*	9	2.1	0.1–4.0	7	3.0	1.2–6.0	16	3.4	2.0–5.5	6	4.2	1.5–8.8	5	5.4	1.8–12.2
*Po*	6	1.4	0.5–3.0	2	0.8	0.1–3.0	5	1.1	0.4–2.5	3	2.1	0.4–6.0	12	13.0	6.9–21.7
*Pm*, *Po*	0	0.0	0–0.9*	0	0.0	0–1.6*	1	0.2	0.0–1.2	0	0	0.0–2.5*	0	0.0	0–3.9*
**Grand total**	**427**	**100**		**236**	**100**		**464**	**100**		**143**	**100**		**92**	**100**	

*Pf*: *P*. *falciparum*; *Pm*: *P*. *malariae*; *Po*: *P*. *ovale* spp.;

* one-sided, 97.5% confidence interval

**Fig 3:**
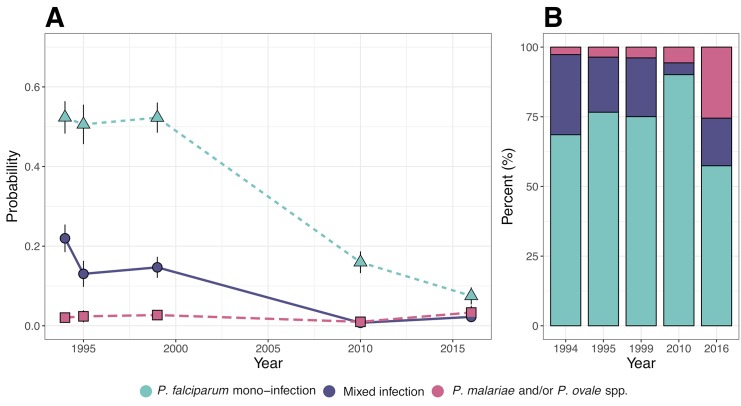
Multinomial logistic model predicted probabilities of *P*. *falciparum* mono-infection, *P*. *falciparum* mixed infection, and non-falciparum, i.e. *P*. *malariae* and/or *P*. *ovale* spp., infection over time. **A)** The model predicted probability of infection over time. Predictions are adjusted for age, sex and fever at the time of survey with all covariates at their respective means. The black error bars denote the 95% confidence interval of the prediction. **B)** Relative contribution of *P*. *falciparum* mono-infections, *P*. *falciparum* mixed infections, and non-falciparum infections to the overall parasite prevalence over time. Estimates are based on the model predicted probabilities presented in **A**.

A multinomial logistic model was used to estimate the relative risk ratio of *P*. *falciparum* mono-infection, *P*. *falciparum* mixed infection and non-falciparum infection compared to being uninfected over time. The adjusted probability (adjusting for age, gender, and fever at time of survey) of being infected with either a *P*. *falciparum* mono-infection, mixed infection, or a non-falciparum infection declined significantly from 1994 to 2010 (Table 4, Fig 3A). With all covariates at their mean value, the model estimated a reduction in the prevalence of *P*. *falciparum* mono-infection from 52.0% to 16.8%, for mixed infections from 22.3% to 0.7%, and for non-falciparum infections from 2.1% to 1.0% (Fig 3A, Table 4). From 2010 to 2016, the model-predicted probability of *P*. *falciparum* mono-infections continued to decline while the probability of both mixed infections and non-falciparum infections increased significantly from 0.7% to 2.1% and 1.0% to 3.3%, respectively (Fig 3A and 3B, Table 4). In the beginning of the study period approximately 90% of *P*. *malariae* and *P*. *ovale* spp. infections were detected as mixed species infections with *P*. *falciparum* (Fig 3B). However, this changed over time towards a greater proportion of these infections occurring as mono-infections. In 2016, 60% of non-falciparum infections were found to occur as mono-infections (Fig 3B, Tables 3 and 4).

**Table 4:** Changes in the relative frequency of *P*. *falciparum* mono- and mixed infections, as well as non-falciparum infections over time evaluated using GEE multinomial logistic regression. Adjusted relative risk ratios (RRR) of mono-infection, mixed, or non-*falciparum* infection each year of survey, relative to being uninfected at base-line in 1994.

	*P*. *falciparum* mono-infection			*P*. *falciparum* mixed infection			non-falciparum infection		
Year	RRR^1^	95% CI	*p*	RRR^1^	95% CI	*p*	RRR^1^	95% CI	*p*
**1994**	Ref.	-	-	Ref.	-	-	Ref.	-	-
**1995**	0.68	0.51–0.91	0.009	0.41	0.27–0.60	<0.001	0.85	0.36–1.98	0.7
**1999**	0.78	0.61–1.01	0.062	0.50	0.36–0.71	<0.001	1.01	0.51–1.97	0.985
**2010**	0.09	0.07–0.12	<0.001	0.01	0.00–0.02	<0.001	0.14	0.06–0.33	<0.001
**2016**	0.04	0.03–0.06	<0.001	0.02	0.01–0.04	<0.001	0.44	0.20–0.96	0.039

^1^ All relative risk ratios are adjusted for age (as a categorical variable in five categories, <5, 5–8, 9–12, 13–16, >16), sex and fever at the time of sampling.

### Symptomatic infections at the time of survey

The number of symptomatic infections occurring at the time of the cross-sectional survey varied over the years and was greater for *P*. *falciparum* mixed and mono-infections compared to non-falciparum infections (S1 Table). The odds of presenting with fever at the time of survey (adjusted for age, sex, and survey year) was estimated to be approximately 4 to 5 times greater if harbouring a *P*. *falciparum* mono-infection (adjusted OR: 4.9, 95% CI 1.45–16.67, *p* = 0.011) or a *P*. *falciparum* mixed infection (adjusted OR: 3.84, 95% CI 1.08–13.57, *p* = 0.036) compared to a *P*. *malariae* and/or *P*. *ovale* spp. infection. There was no significant difference in the odds of presenting with fever at the time of survey between those infected with *P*. *malariae* and/or *P*. *ovale* spp. and those who were PCR negative (adjusted OR: 1.54, 95% CI: 0.45–5.19, *p* = 0.49).

## Discussion

In the present study we assessed the prevalence of *Plasmodium* spp. in five cross-sectional surveys over two decades in a Tanzanian village experiencing a substantial reduction in the prevalence of *P*. *falciparum* infection. We used real-time PCR to obtain a high sensitivity and specificity in detection of both mixed-species and non-falciparum infections. *Plasmodium malariae* and *P*. *ovale* spp., but no *P*. *vivax*, infections were detected throughout the study as both mixed and mono-infections. Although the prevalence of all species declined over time, the decline in *P*. *ovale* spp. prevalence was smaller leading to a relative increase in the number of infections being due to *P*. *ovale* spp. Furthermore, there was a shift of *P*. *malariae* and *P*. *ovale* spp. infections from occurring almost exclusively as mixed species infections with *P*. *falciparum* to occur more commonly as mono-infections. This illustrates that the transmission patterns of non-falciparum species do not necessarily follow those of *P*. *falciparum*. These findings emphasise the need to carefully monitor the prevalence and transmission trends of non-falciparum species of *Plasmodium* to improve our understanding of their epidemiology and to guide specific interventions aimed at achieving malaria control and elimination.

Previous studies of the Nyamisati cohort have examined the changing transmission intensity of *P*. *falciparum* between 1985 and 2010 [31,33]. Here, the expansion of the analysis to non-falciparum species revealed a parallel reduction in the odds of infection of 93% for *P*. *falciparum* and 94% for *P*. *malariae*, and *P*. *ovale* spp., between 1994 and 2010. We then observed a further 46% reduction in the odds of *P*. *falciparum* infection until 2016. In contrast, the odds of *P*. *malariae* and *P*. *ovale* spp. infection increased by 2-fold and 6-fold, respectively, from 2010 to 2016. The observed increase in the relative contribution of non-falciparum infections to the overall prevalence of infection is in line with reports from Burkina Faso where an increase in the prevalence of *P*. *malariae* infection was observed by microscopy as transmission of *P*. *falciparum* decreased [37]. However, the data is somewhat contrasted by the findings from Dielmo, Senegal, of a near elimination of *P*. *malariae* and *P*. *ovale* spp. between 1990 and 2010 when the prevalence of *P*. *falciparum* decreased [38]. Although this longitudinal study [38] used only microscopy for parasite detection, the near absence of *P*. *malariae* and *P*. *ovale* spp. in Dielmo has later been confirmed using PCR [39]. These differences between geographical sites highlight the need to obtain local estimates of the transmission patterns of all *Plasmodium* species.

The reduction in the prevalence of *P*. *falciparum* in Nyamisati between 1985 and 2010 has been attributed to the presence of a research and healthcare team who provided prompt access to diagnosis and treatment, more effective antimalarial treatment (i.e. ACTs), and vector control measures (ITNs were distributed after the surveys in 1993 and 1999) [31,33]. LLINs were distributed to all survey participants after the survey in 2010. This might have contributed to the further decline in *P*. *falciparum* prevalence observed after 2010. However, it does not appear to have affected the prevalence of *P*. *malariae* and *P*. *ovale* spp. to the same extent. In 2016, *P*. *ovale* spp. superseded *P*. *malariae* as the second most commonly detected species and its prevalence returned to levels similar to those in 1999, i.e. prior to any large-scale intervention with bed nets at the study site [31].

In Tanzania, all *Plasmodium* species appear to share the same primary malaria vectors [40]. Entomological data are not available from the study site but according to previous entomological studies in the Rufiji delta area, the important primary malaria vectors are members of the *An*. *gambiae* complex (e.g. *An*. *gambiae* ss, *An*. *arabiensis* and *An*. *merus*), all of which are highly anthropophilic and predominantly indoor-biting at night [41,42]. Accumulating evidence suggest that large-scale distribution of LLINs affects both the behaviour and composition of vector populations, making secondary vectors, which are prone to outdoor biting, more important for malaria transmission [40,43,44]. Specific changes in vector populations could in theory affect the transmission of each *Plasmodium* species differently, but whether *P*. *malariae* and *P*. *ovale* spp. are more or less efficiently transmitted by the secondary vectors compared to *P*. *falciparum* is currently unknown.

According to current WHO guidelines, primaquine treatment is recommended to prevent relapses of *P*. *ovale* spp. infections [45]. However, to our knowledge, primaquine has not been used in the village. The absence of relapse prevention, which is likely to be required in order to eliminate *P*. *ovale* spp., could theoretically contribute to a lower relative reduction in transmission of *P*. *ovale* spp. compared to the other species. With the available data, it is not possible to determine whether this could explain the observed transmission patterns in Nyamisati. The first-line antimalarial treatment used at the study site did not differ depending on *Plasmodium* species but changed during the study period from SP to ACT when ACT became readily available in the village in 2009 [31]. As for *P*. *falciparum*, ACTs are highly efficacious against asexual stages of both *P*. *malariae* and *P*. *ovale* spp. and the change of first-line anti-malarial is unlikely to have contributed to the smaller relative reduction in non-*falciparum* infections [46,47].

In sub-Saharan Africa, a majority of infections with *P*. *malariae* and *P*. *ovale spp*. are reported to occur as mixed species infections with *P*. *falciparum* [7,48–50]. Although a vast majority (approximately 90%) of non-falciparum infections occurred as mixed species infections during the early years of the study, this changed over time. At the end of the study period, approximately 31% of *P*. *malariae* and 57% of *P*. *ovale spp*. infections occurred as mono-infections. Furthermore, our data indicate that individuals harbouring non-*falciparum* infections are less likely to be symptomatic and thereby may be less likely to seek medical treatment. The observed shift has important implications for malaria control and monitoring of transmission intensity. It increases the importance of accurately identifying each species independently and highlights the need to detect and actively target asymptomatic carriers of infection in order to provide interventions that can reduce the transmission of non-falciparum malaria.

The present study is somewhat limited by the repeated cross-sectional design as well as the relatively long time-intervals between the surveys. However, a substantial number of individuals participated in multiple surveys, providing a longitudinal aspect of the study design. An even closer follow-up on the individual level may have provided a more detailed understanding of the epidemiology of non-falciparum infections. To account for annual variation in the start of the peak transmission season, all surveys were conducted during the beginning of the long rainy season (March-May, depending on year) [31]. During late 2015 and early 2016, the coastal regions of Tanzania were heavily affected by an El Niño Southern oscillation which lead to greater than average rainfall in the Rufiji area until mid-February 2016, as well as greater than average temperatures and humidity during the following months [51]. This likely increased both the Anopheles vector density and the rate of parasite development within the vector and thus the potential for malaria transmission [51].

Another limitation of the study is that the PCR-method used does not distinguish between the two recently described sympatric species of *P*. *ovale* (*P*. *ovale curtisi* and *P*. *ovale wallikeri*) [15,34]. Although the real-time PCR sensitively detects both, we were unable to examine whether both species are endemic in this area and how their relative frequencies might have changed over time.

Because molecular methods are still expensive and often difficult to implement in large scale for routine surveillance, detection of *Plasmodium* infection relies largely on the use of microscopy and/or RDTs that lack sensitivity for the detection *P*. *malariae* and *P*. *ovale* spp. [29,52,53]. Data from Kenya suggests that as much as 50% of *P*. *malariae* infections may occur as sub-microscopic infections [29]. In addition, current WHO guidelines regarding the selection and procuration of RDTs are based on the assumption that a vast majority of non-falciparum infections occur as mixed species infections [49,54]. The guidelines state that RDTs based only on the detection of *P*. *falciparum* histidine rich protein (HRP-2) are sufficient in most areas of sub-Saharan Africa [49,54,55]. The issue of using a *P*. *falciparum* HRP-2-only test, which by design cannot detect non-falciparum infections, has recently been recognised as a problem for diagnosis and surveillance in Senegal where *P*. *malariae* and *P*. *ovale* spp. have also been reported to occur more frequently as mono-infections [56].

Our findings highlight some of the key challenges that will need to be addressed if malaria elimination is to be achieved. The observed increase in the prevalence of *P*. *malaria* and *P*. *ovale* spp. that occurred while the prevalence of *P*. *falciparum* declined may support previously raised concerns that strategies designed for reducing transmission of *P*. *falciparum* may be less effective in reducing transmission of the non-falciparum species of *Plasmodium* [5,57,58]. For *P*. *malariae* and *P*. *ovale* spp., this is likely due to their species-specific ability to cause persistent asymptomatic infections in combination with a low effectiveness of current diagnostic and surveillance tools which contribute to their resilience to interventions [5]. In order to further limit malaria transmission, it is of utmost importance to be able to identify and target asymptomatic carriers of infection, not only for *P*. *falciparum* but also for *P*. *malariae* and *P*. *ovale* spp. where asymptomatic carriage appears to be even more common [24,38,59]. There is a pressing need for easy-to-implement, cost-effective tools for diagnosis and surveillance (e.g. species-specific RDTs) that can sensitively and accurately detect all species. This could be further improved by the development of reliable species-specific serological tools that can be used to monitor exposure [33,60].

In summary, we observed the maintenance of *P*. *ovale* spp., and to a lesser extent of *P*. *malariae*, infections despite a substantial and continuous reduction in the prevalence of *P*. *falciparum* over a period of 22-years. This demonstrates that the transmission patterns of non-*falciparum* species do not necessarily follow those of *P*. *falciparum*, stressing the need for attention towards *P*. *malariae* and *P*. *ovale* spp. transmission in Africa. Furthermore, the prevalence patterns observed by PCR highlight the need for field-applicable tools to detect non-falciparum infections. Malaria elimination will require a better understanding of the specific epidemiological features of *P*. *malariae* and *P*. *ovale* spp. as well as improved tools for efficient monitoring of all *Plasmodium* species, with a particular focus towards identifying asymptomatic carriers of infection and designing appropriate intervention strategies to reach the goals of elimination.

## Supporting information

S1 Table The total and number of symptomatic *P*. *falciparum* mono-infections, mixed and non-*falciparum* infections detected each year of survey. (DOCX)

S1 Data Individual-level data on the presence of *Plasmodium* infection determined using species-specific real-time PCR. (XLSX)

S1 Checklist STROBE statement. (DOC)
