# Supplementary material for: Persistent transmission of Plasmodium malariae and Plasmodium ovale species in an area of declining Plasmodium falciparum transmission in eastern Tanzania
Source: PLoS Negl Trop Dis. 2019 May 28;13(5):e0007414. doi: 10.1371/journal.pntd.0007414 (PMC6555537; doi:10.1371/journal.pntd.0007414)
Supplement: S1 Table — (DOCX) [file pntd.0007414.s001.docx]

**S1 Table.** The **t**otal and number of symptomatic *P. falciparum* mono-infections, mixed and non-*falciparum* infections detected each year of survey.

|  |  |  |  |  |  |
| --- | --- | --- | --- | --- | --- |
|  | **1994** | **1995** | **1999** | **2010** | **2016** |
|  |  |  |  |  |  |
|  |  |  |  |  |  |
| ***P. falciparum* mono-infections, n** | 289 | 177 | 345 | 128 | 57 |
| symptomatic, n (%) | 22 (8) | 30 (17) | 74 (21) | 8 (6.0) | 14 (25) |
|  |  |  |  |  |  |
|  |  |  |  |  |  |
| ***P. falciparum* mixed species infections, n** | 123 | 50 | 97 | 6 | 18 |
| symptomatic, n (%) | 7 (6) | 6 (12) | 23 (24) | 0 (0) | 3 (17) |
|  |  |  |  |  |  |
|  |  |  |  |  |  |
| **Non-*falciparum* infections, n** | 15 | 9 | 22 | 9 | 17 |
| symptomatic, n (%) | 1 (6) | 1 (11) | 0 (0) | 0 (0) | 1 (6) |
|  |  |  |  |  |  |
